# Skin-cancer screening preferences and trust in clinicians among outdoor enthusiasts: preference for specialist-led checks

**DOI:** 10.7717/peerj.21222

**Published:** 2026-06-08

**Authors:** Mike Climstein, Michael Stapelberg, Ian J. Miller, Nedeljka Rosic, Jeremy Hudson, Paul Coxon, Nathaniel Milani, Chris Robinson, Joe Walsh

**Affiliations:** 1 Aquatic Based Research, Faculty of Health, Southern Cross University, Bilinga, Queensland, Australia; 2 Exercise and Sport Science Exercise, Health & Performance Faculty Research Group Faculty of Health Sciences, University of Sydney, Sydney, New South Wales, Australia; 3 Skin Clinic, Robina, Queensland, Australia; 4 Biomedical Sciences, Faculty of Health, Southern Cross University, Bilinga, Queensland, Australia; 5 North Queensland Skin Centre, Townsville, Queensland, Australia; 6 Princess Alexandra Hospital, Woolloongabba, Queensland, Australia; 7 Melanoma Scan, Warner, Queensland, Australia; 8 Faculty of Science and Engineering, Southern Cross University, Bilinga, Queensland, Australia; 9 Sport Science Institute, Sydney, New South Wales, Australia; 10 AI Consulting Group, Sydney, New South Wales, Australia

**Keywords:** Skin neoplasms, Early detection, Patient preference, Primary health care, Early detection of skin cancer, Basal cell carcinoma, Squamous cell carcinoma, Melanoma

## Abstract

**Introduction:**

Australian outdoor enthusiasts are at heightened risk of skin cancer due to high levels of ultraviolet (UV) exposure. Regular screening is essential for early detection; little is known about how trust in clinician expertise and screening frequency shape behaviors in this cohort .

**Methods:**

A cross-sectional survey of 1,403 adult outdoor enthusiasts attending a skin cancer clinic collected data on UV exposure, screening habits, clinician preference and time since last skin check. Group comparisons and multivariable logistic regression identified predictors of specialist preference, and screening intervals were correlated with cancer detection.

**Results:**

Over half of participants (54.9%) performed regular self/partner skin checks, while 7.8% had never had a clinical check. Trust in clinician expertise was strongly influential, with 84.2% prioritising who performed the check, 63.6% most recently screened by a skin-cancer specialist. Multivariable analysis, a prior skin cancer diagnosis (OR = 2.13, *p* < 0.001), regular self-examination (OR = 1.61, *p* = 0.002), and sunscreen use (OR = 1.49, *p* = 0.047) independently predicted specialist preference. Age, gender, skin type, family history, cumulative UV exposure, and activity were not significant predictors. Cancer diagnoses, including melanoma, were most common among those screened within the past 12–24 months, declining sharply beyond two years.

**Conclusion:**

Prior skin cancer experience and proactive protective behaviors strongly influenced specialist preference. The temporal distribution of diagnoses observed in this cohort was compatible with the potential value of regular screening among UV-exposed outdoor enthusiasts; however, causal inference cannot be drawn from this cross-sectional design.

## Introduction

Australia carries one of the world’s heaviest skin-cancer burdens (Cancer Council 2023a; Cancer Council 2023b) with more than two-thirds of Australians projected to be diagnosed with a skin cancer in their lifetime. In 2023, Medicare data recorded over one million treatments for keratinocyte cancers, also known as non-melanoma skin cancer, and not melanoma. keratinocyte cancer predominantly include basal cell carcinoma (BCC) and squamous cell carcinoma (SCC), with other variants being rare and of limited clinical relevance (Cancer Council 2023a; Cancer Council 2023b). The economic impact of skin cancer in Australia is substantial: recent estimates place annual health-system spending on skin cancers at approximately A$1.7–1.9 billion, making skin cancer the most expensive cancer group to manage in Australia (Cancer Council 2023a; Cancer Council 2023b). Although BCC or SCC account for significantly more cases than melanoma, melanoma causes the majority of skin cancer-related deaths, with approximately 1,400 reported in 2024 (Australian Government Cancer Australia, 2025). The Australasian College of Dermatologists also reports persistently high mortality and emphasizes the ongoing public health burden of skin cancer (Australasian College of Dermatologists, 2024a).

Current Australian recommendations prioritize targeted, not population-wide, screening for skin cancer (Janda et al. 2020). The Royal Australian College of General Practitioners (RACGP) recommends opportunistic clinical skin examinations for people at above-average risk (fair complexions that burn easily, light eyes, light/red hair, family history of skin cancer and male gender) (Royal Australian College of General Practitioners 2025a; Royal Australian College of General Practitioners, 2025b). The RACGP recommends regular skin checks, at least annually, for those at high risk, including familial or personal history of skin cancer, increased numbers of moles/naevi or immunocompromised (Royal Australian College of General Practitioners 2025a; Royal Australian College of General Practitioners, 2025b; Johns et al., 2024). The Cancer Council’s early detection position statement similarly advises clinical skin examinations every six to 12 months for higher-risk individuals, alongside education on the importance of regular self-examination (Cancer Council 2023a; Cancer Council 2023b). The Australasian College of Dermatologists recently stated that it does not recommend population-based melanoma screening because the mortality benefit has not been demonstrated (Australasian College of Dermatologists, 2024a). Together, these statements reflect a consensus toward opportunistic and risk-stratified approaches in both primary care and specialty settings (Sinclair, 2012; Arasu, Meah & Sinclair, 2019).

Previous studies have examined differences in diagnostic patterns across clinician types in skin cancer detection. These studies are often cited in discussions of provider expertise and may influence patient perceptions of clinician capability. In a landmark Australian study comparing general practitioners (GPs) with dedicated primary-care skin cancer doctors, sensitivities for BCC were 0.79 (GPs) and 0.89 for skin cancer doctors; SCC were 0.69 (GPs) and 0.67 (skin cancer doctors); and melanoma (small numbers noted in the study) were 0.29 (GPs) and 0.60 (skin cancer doctors) (Youl et al., 2007). For keratinocyte cancers combined, sensitivities exceeded 0.90 in both groups (Youl et al., 2007). An earlier Australian study in older adults found that GPs could achieve high melanoma screening sensitivity but at the cost of lower specificity and positive predictive value, thus highlighting the trade-off between missing melanomas and excising benign lesions (Burton et al., 1998). Contemporary primary-care reviews reaffirm that Australian GPs are generally accurate in diagnosing keratinocyte cancers; while varied performance was observed when clinicians were tasked with distinguishing early-stage melanoma from benign pigmented lesions (Emery, 2011). In specialised primary-care clinics, high sensitivity was reported for the excision of melanoma from suspicious pigmented lesions, again with lower specificity (*i.e.,* more benign excisions) as the price of caution (Green et al., 2021). Dermatology-led settings typically show lower “number-needed-to-biopsy” for melanoma than general practice, consistent with higher specificity in specialist hands, although estimates vary across studies and jurisdictions (Wilkinson, Askew & Dixon, 2006). Recent Australian data also shows that the vast majority of melanomas (approximately 77%) are diagnosed and initially managed in primary care clinics, underscoring the central role of GPs and skin-cancer doctors in early detection (Pandeya et al., 2023). Importantly, these studies evaluated diagnostic performance in specific clinical settings and should not be interpreted as evidence that one provider group is inherently superior to another. Rather, such findings may contribute to patient perceptions of expertise, which in turn influence screening preferences. The present study does not assess diagnostic accuracy but instead evaluates perceived trust and provider preference among patients.

Self-skin examination is a widely promoted early-detection behavior in Australia, with national data suggesting that a substantial proportion of adults engage in some form of routine self-checking. It can be performed using the ABCD rule, which helps distinguish benign melanocytic nevi from suspicious melanomas (Hauschild et al., 2011). The 2013–14 National Health Survey (Australian Bureau of Statistics, 2015), which included more than 19,000 Australians, reported that approximately two-thirds (66%) regularly checked their skin for changes in freckles or moles. Similarly, a 2017 survey by The Skin and Cancer Foundation found that 70% of women and 64% of men self-examined their skin for signs of disease, including skin cancer (Skin & Cancer Foundation Inc., 2017). These population-level behaviors establish an important benchmark against which cohort-specific self-screening patterns can be interpreted.

Aquatic and non-aquatic outdoor enthusiasts experience fundamentally different ultraviolet (UV)-exposure profiles (Snyder et al., 2020). Variations in people’s natural skin protection (*e.g.*, depending on the pigment melanin levels), the use of safety measures (*e.g.*, quality of sunscreens and/or use of protective clothing) may influence both their biological risk for skin cancer and their screening behavior. Aquatic activities such as surfing and swimming expose participants to direct UV radiation, reflection from the water surface, and prolonged exposure due to minimal photoprotective clothing, contributing to high intermittent UV doses—an established pathway for melanoma, BCC and SCC development. Prior work in Australian surfers and swimmers demonstrated markedly elevated rates of actinic keratoses, keratinocyte cancers and melanoma, including point-prevalence estimates up to 76-fold higher for melanoma compared with the general population (Climstein et al., 2022). Similarly, lifetime-prevalence data in Australian surfers showed increased risks across all major skin cancer types, with lesions commonly located on high-exposure sites such as the face, back and arms (Climstein et al., 2016). By contrast, non-aquatic outdoor activities (*e.g.*, walking, running, cycling) involve more variable clothing coverage, less reflective UV exposure and different immersion patterns, which may alter both the anatomical distribution and cumulative dose of UV exposure. These biologically and behaviorally distinct exposure profiles justify examining aquatic and non-aquatic groups separately, as they may differ in risk perception, photoprotection habits, screening uptake and trust in clinician expertise.

Despite the clinical salience of “skin checks” among Australians with high cumulative UV exposure, little is known about how patients’ preferences for provider type (GP, primary care skin cancer doctor, dermatologist) or if a prior skin cancer diagnosis influence real world screening behavior, especially in outdoor aquatic and non-aquatic enthusiasts (Miller et al., 2023; Climstein et al., 2024; Melanoma Institute Australia, 2024). Understanding how perceived expertise and trust in different clinician types influence screening behaviors and preferences is important for understanding patient pathways within Australia’s risk-stratified screening landscape.

Accordingly, this study aimed to (i) quantify outdoor enthusiasts’ screening preferences and trust in different clinician types (GPs, primary care skin cancer doctors, dermatologists), (ii) examine how these preferences relate to screening behavior and prior skin cancer diagnoses, and (iii) explore differences between aquatic and non-aquatic outdoor enthusiasts. By linking stated preferences to actions and outcomes, we seek to inform pragmatic, targeted early-detection strategies within Australia’s risk-stratified screening landscape (Royal Australian College of General Practitioners 2025a; Royal Australian College of General Practitioners, 2025b).

## Methods

### Study design and setting

The Human Research Ethics Committee at Southern Cross University approved this study on May 11th, 2020 (2020/47). All participants provided written, informed consent. This study was a cross-sectional survey of Australian adults who regularly participated in outdoor physical activity, recreation or sport and who attended a skin cancer clinic for a skin examination. The structure and reporting follow our previously published methods (STROBE aligned) (Miller et al., 2023), in which participants completed a standardized questionnaire capturing demographics, sun-exposure behaviors, photoprotection and skin cancer history. This survey was administered in a clinic setting; all data were collected through a survey followed by a subsequent clinical examination. The focus for this study was on participant preferences for skin cancer screening provider and trust in clinician expertise. The questionnaire domains and item framing were adapted verbatim, where applicable, from our earlier instruments to preserve comparability across cohorts (Climstein et al., 2024).

Although participants were recruited in a dedicated primary-care skin cancer clinic, the survey captured retrospective behaviors and screening histories occurring across multiple settings, including general practice, dermatology and plastic-surgery clinics. This design allowed us to examine participants’ real-world screening patterns independent of the recruitment site.

Given the cross-sectional design, the study examines associations between screening interval and cancer detection at a single time point. It does not permit the determination of temporal directionality or causal relationships between screening frequency and diagnostic outcomes.

### Participants and recruitment

Participants were asked to identify the outdoor physical, recreational, or sporting activity in which they participated for a minimum of ≥ one hour/week. Activities were categorized as aquatic and non-aquatic. Aquatic participants primarily engaged in activities such as surfing and swimming, while the non-aquatic group included individuals who regularly participated in activities like walking, running or cycling. Recruitment used the same broad-access approach we employed previously (local media (TV, radio, print) and community networks) (Climstein et al., 2022; Miller et al., 2023; Climstein et al., 2024). This approach mirrors the inclusive recruitment strategy used in our prior studies that enrolled community-based outdoor enthusiasts (Climstein et al., 2022; Miller et al., 2023; Climstein et al., 2024).

### Survey instrument and variables

The questionnaire consisted of four sections modelled on our prior instruments and included the following: (i) demographics (age, gender), (ii) activity profile (primary physical activity/sport, weekly outdoor hours, proportion of time during peak UV periods), (iii) photoprotection (sunscreen use, clothing), and (iv) skin cancer risk/history (Fitzpatrick skin type, family history of skin cancer, prior personal diagnosis). In this study, ‘UV-exposed hours’ refers to the total number of hours participants reported spending outdoors in direct sunlight across both recreational (*e.g.*, surfing, swimming, stand up paddleboarding, walking, running, cycling) and occupational settings. This definition aligns with prior exposure-assessment approaches in outdoor and sporting cohorts (Hammond, Reeder & Gray, 2009;  Downs et al., 2020), in which cumulative occupational and recreational sunlight hours are combined to provide a more comprehensive estimate of total UV burden

### Primary outcomes

The primary outcome variables in this study were trust in clinician expertise, measured using the survey item: “When you get a skin check, do you prefer a specialist (dermatologist or dedicated skin-cancer doctor) because you trust their expertise more than a general practitioner?”. For clarity, in this study, the term “skin cancer doctor” reflects participant self-report and refers to a GP whom the participant perceived to have a specific interest or expertise in skin cancer. This is not a formally protected or standardized medical designation and does not represent verified specialist accreditation. The category therefore reflects patient perception of provider expertise rather than an objective assessment of training or credentialing.

The key independent variables included age, gender, Fitzpatrick skin type, family history of skin cancer, self and/or partner skin examination, regular sunscreen use, weekly UV-exposed hours, previous skin cancer diagnosis, and physical activity/exercise type (aquatic *vs* non-aquatic). These variables were selected based upon established determinants of skin cancer risk and screening behavior in prior literature, as well as consistency with our previous studies, to ensure construct validity and facilitate cross-study comparisons (Climstein et al., 2022; Miller et al., 2023).

### Statistical analysis

All analyses were planned *a priori*. The dataset underwent initial screening to ensure completeness and to identify any implausible entries. Continuous variables were evaluated for normality using both statistical (Shapiro–Wilk and Kolmogorov–Smirnov tests with Lilliefors correction) and graphical methods, including Q–Q plots, as well as by examining skewness and kurtosis values. Homogeneity of variances was tested using Levene’s test to evaluate potential heteroscedasticity.

Descriptive statistics for continuous variables are reported as mean ± standard deviation (SD), while categorical variables are expressed as counts and percentages, aligning with our prior methodological reporting.

### Group comparisons

Between-group differences (aquatic *vs* non-aquatic) used Pearson’s *χ*^2^ tests for categorical variables and independent-samples *t*-tests for normally distributed continuous variables; when normality was violated, Mann–Whitney U tests were applied. A two-tailed *α* was set at 0.05 to determine statistical significance.

### Predictors of trust in specialists

We modelled trust in clinician expertise as a binary outcome using logistic regression with simultaneous entry of covariates: age, gender, Fitzpatrick skin type, family history of skin cancer, personal history of skin cancer, self and/or partner exam, sunscreen use, UV hours and activity type. The included behavioral predictors (self/partner examination and sunscreen use) were conceptually distinct variables entered simultaneously; inspection of regression coefficients and standard errors indicated no evidence of instability suggestive of problematic multicollinearity. Our model diagnostics included assessment of calibration using Hosmer–Lemeshow goodness-of-fit test and explanatory power was summarized using Nagelkerke R^2^ (Bewick, Cheek & Ball, 2005). All analyses and visualizations were conducted in SPSS (v31.0) and R (version v4.4.1; R Core Team, 2024), using an analytical toolchain consistent with previous methodologies described in the literature.

### Results

### Demographic characteristics of participants

**Figure 1:**
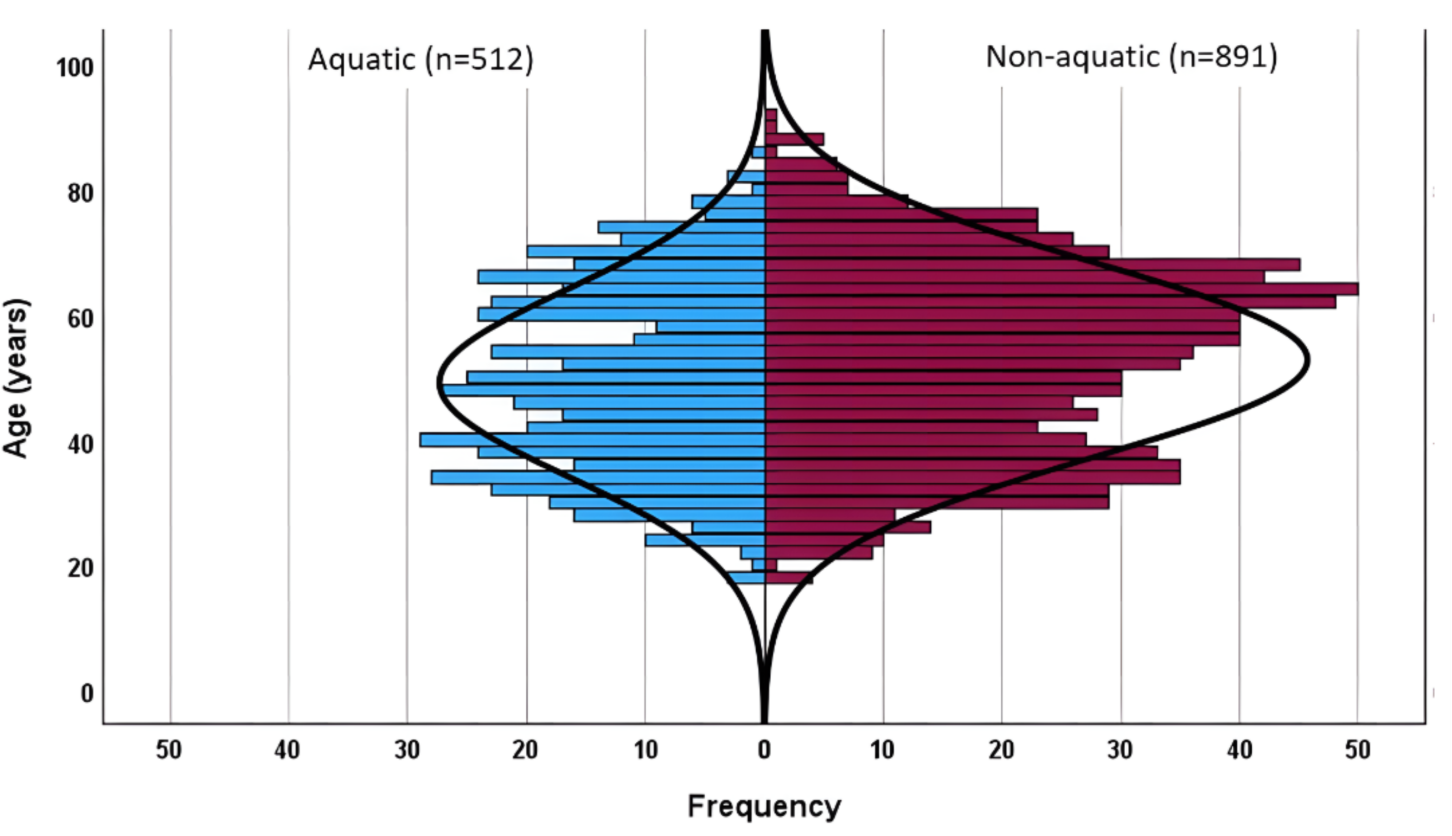
Population pyramid (with line of normality).

A total of 1,403 participants attended the clinic for a skin cancer screening and consented to participate in the study (Fig. 1). There were no adverse events associated with the survey or subsequent skin examination. The majority of participants were female (52.7%) and were non-aquatic enthusiasts (63.5%). Non-aquatic participants were significantly older (+7.3%) than aquatic participants; however, aquatic participants were significantly taller (+3.2%) and had a significantly higher mass (+6.3%). Body mass index (BMI) did not differ significantly between groups, and the majority of participants fell within the World Health Organisation’s normal BMI range (≥18.5 to ≤24.9 kg/m^2^) (World Health Organization, 2018) (Table 1).

**Table 1:** Participants’ demographics Values are mean (±SD), number and per cent (n, %), and 95% confidence intervals (95% CI).

**Parameter**	**Group** (*n* = 1,403)	**Aquatic** (*n* = 512)	**Non-aquatic** (*n* = 891)	***P*-value**
Primary Activity • Surfing • Swimming • SUP • Walk/run • Cycle	–	• Surfing 310 (22.1) • Swim 161 (11.5) • SUP 41 (2.9)	• Walk/run 877 (62.5) • Cycle 12 (0.9)	–
Age (years)	51.4 (15.4) [50.6–52.3]	49.1 (14.9) [47.8–50.4]	52.7 (15.6) [51.7–53.8]	<0.001
Gender (n, %)				
Males	664 (47.3)	351 (68.5)	313 (35.1)	<0.001
Females	739 (52.7)	161 (31.5)	578 (64.9)	<0.001
Height (cm)	171.6 (9.9) [171.1–172.2]	175.1 (9.7) [174.2–175.9]	169.7 (9.6) [169.0–170.3]	<0.001
Mass (kg)	76.1 (15.4) [75.3–76.9]	79.1 (13.8) [77.9–80.3]	74.4 (16.0) [73.3–75.4]	<0.001
BMI (kg/m^2^) Category (n, %) • underweight • normal • overweight • obese	25.7 (4.3) [25.5–25.9] 77 (5.5) 612 (43.6) 517 (36.8) 196 (14.0)	25.7 (3.7) [25.4–26.1] 14 (2.7) 229 (44.7) 220 (43.0) 49 (9.5)	25.7 (4.6) [25.4–26.1] 63 (7.1) 383 (42.9) 297 (33.3) 147 (16.5)	0.981

**Notes.**

Where: SUP, stand-up paddle boarders; BMI, body mass index.

 Non-aquatic participants reported significantly more activity hours for the previous year (+23.1%); however, significantly more aquatic participants reported completing their activity during peak UV and for a higher percentage of time (+57.3%, relative). Aquatic participants also reported greater occupational UV exposure as compared to non-aquatic participants (+65.7%). The majority of participants were identified as Fitzpatrick skin types II and III, with no type I, V or VI identified in either aquatic or non-aquatic groups. This distribution likely reflects the demographic characteristics of the local population and the clinic-attending cohort rather than the absence of these skin types within the broader Australian community.

Approximately 40% of participants reported having a family (first-degree relative) history of malignant melanoma and approximately 10% of participants reported having a family history of a keratinocyte cancers (BCC or SCC). A similar percentage of participants reported a personal history of skin cancer; the majority were BCC, followed by SCC and melanoma. The majority (76.4%) of participants reported having a history of blistering sunburns during their childhood. With regard to photoprotection behaviors, the majority (85.3%) of participants reported using sunscreen. However, significantly more aquatic participants reported using a combination of sunscreen and zinc (Table 2).

**Table 2:** Participants’ sun-exposure patterns and skin cancer risk indicators. Values are mean (±SD), number and per cent (n,%), and 95% confidence intervals (95% CI).

**Parameter**	**Group** (*n* = 1,403)	**Aquatic** (*n* = 512)	**Non-aquatic** (*n* = 891)	***P*-value**
Total activity hours per year (hrs/yr)	236.8 (247) [223–249]	206.5 (272) [180–226]	254.2 (230) [239–270]	<0.001
Estimated lifetime UVR exposure (hrs)	8454 (11757) [7,847–9,094]	6543 (11874) [5,511–7,603]	9565 (11551) [8,804–10,341]	<0.001
Activity during peak UVR (yes, n, %)	922 (65.7)	431 (84.2)	491 (55.1)	<0.001
Activity completed during peak UVR (%)	27.4 (30.7) [25.6–28.9]	35.7 (30.5) [33.1–38.5]	22.7 (29.8) [20.3–24.3]	<0.001
Total work activity with UVR exposure (hrs/yr)	303.9 (666) [269–340]	418.4 (730) [355–483]	238.5 (618) [198–279]	<0.001
Fitzpatrick skin type				0.012
• skin type I • skin type II • skin type III • skin type IV • skin type V • skin type VI	0 1093 303 7 0 0	0 380 131 1 0 0	0 713 172 6 0 0	
Family history of skin cancer (yes, %)				
• melanoma • keratinocyte cancer	571 (40.7) 146 (10.4)	223 (43.5) 44 (8.6)	348 (39.1) 102 (11.4)	0.579
Personal history of skin cancer				
• yes • AK • BCC • SCC • SCC *in situ* • melanoma	602 (42.9) 130 (9.3) 426 (30.4) 182 (13.0) 56 (4.0) 171 (12.2)	222 (43.3) 55 (10.7) 160 (31.3) 73 (14.2) 21 (4.1) 64 (12.5)	380 (42.6) 75 (8.4) 266 (29.9) 109 (12.2) 35 (3.9) 107 (12.0)	0.796 0.266 0.684 0.277 0.973 0.787
History of blistering sunburn (yes, %)	1,072 (76.4)	396 (77.3)	676 (75.9)	0.531
Sunscreen use (yes, %) • reapplied as directed Uses sunscreen and zinc	1,197 (85.3) 675 (48.1) 452 (32.2)	456 (89.0) 275 (60.3) 272 (53.1)	741 (83.2) 400 (54.0) 180 (20.2)	0.003 0.001 <0.001
Wear a hat (yes, %) Wear a rashie (yes, %)	965 (68.8) 1,177 (83.9)	189 (36.9) 352 (68.8)	776 (87.1) 825 (92.6)	<0.001 <0.001

**Notes.**

Where: AK, actinic keratosis; BCC, basal cell carcinoma; SCC, squamous cell carcinoma; UVR, ultraviolet radiation.

### Skin-check practices and trust in clinician expertise

Over half of participants (54.9%) reported routinely conducting self-examinations or partner-assisted checks for suspicious skin lesions. However, a noteworthy 7.8% had never undergone a professional skin check. This finding was similar across both aquatic and non-aquatic participants (*p* = 0.265), indicating comparable rates of self-examination behavior between groups (Table 3).

**Table 3:** Participants’ skin-check practices: self-exams, timing and clinician. Values are mean (± SD), number and per cent (n,%), and 95% confidence intervals (95% CI).

**Parameter**	**Group** (*n* = 1,403)	**Aquatic** (*n* = 512)	**Non-aquatic** (*n* = 891)	***P*-value**
Conduct regular partner and/or self- exam (yes, %)	770 (54.9)	291	479	0.265
When last skin check (n, %)				0.096
• never • <6 months • 1 year • 2 years • 3 years • 4 years • 5 years • >5 years	109 (7.8) 430 (30.6) 449 (32.0) 209 (14.9) 68 (4.8) 24 (1.7) 31 (2.2) 83 (5.9)	35 174 158 74 24 10 16 21	74 256 291 135 44 14 15 62	
^**^Significance of who completed skin check (yes, %)	1,182 (84.2)	437 (37.0)	745 (63.0)	0.390
Who conducted last skin check (n, %)				0.340
• GP • Skin cancer doctor • Dermatologist • Plastic surgeon	293 (20.9) 892 (63.6) 101 (7.2) 8 (0.6)	115 (8.9) 320 (24.7) 37 (2.9) 5 (0.03)	178 (13.8) 572 (44.2) 64 (4.9) 3 (0.02)	

**Notes.**

Where: GP, general practitioner.

** *n* = 1,294 participants.

In terms of the recency of clinician-led skin checks, 32.0% (449 out of 1,403) of all respondents had been screened within the past year, with an additional 30.6% (430 out of 1,403) reporting a check within the past six months. However, nearly 6% (83 out of 1,403) of all participants had not undergone a skin check in over five years, and 7.8% (109 out of 1,403) reported no previous clinical screening.

Trust in clinician expertise appeared to be a dominant behavioral driver: 84.2% affirmed the importance of who performs the skin check. This preference did not differ significantly between activity types (*p* = 0.390), implying a shared recognition among outdoor enthusiasts of the nuanced role of provider expertise in skin cancer detection. Consistent with this, the most commonly cited provider for the last skin check was a dedicated skin cancer doctor (63.6%), far surpassing general practitioners (20.9%), dermatologists (7.2%), or plastic surgeons (0.6%) (Table 3). Importantly, 36.4% of participants reported that their previous skin check had been performed outside of a skin cancer clinic, demonstrating that provider utilization in this cohort reflect varied prior screening behavior rather than an artefact of the clinic-based recruitment. Interestingly, although non-aquatic participants reported lower peak UV exposure (Table 2), they were proportionately more likely to be overdue for screening (>five years since last check), indicating a possible behavioral gap where those with lower perceived exposure may have reduced screening motivation despite actual risk.

### Skin-cancer diagnoses by time since last clinical screening

Across all cancer types, the distribution of confirmed diagnoses showed a clear temporal gradient relative to the recency of the most recent clinician-performed skin check. Actinic keratoses (AK), were the most frequently identified skin lesion type, demonstrating the highest number of positive diagnoses among participants screened within the previous 12 months, with cases peaking at both the <six-month and one-year timepoints before declining steadily thereafter. A similar, though less pronounced, pattern was observed for BCC, which showed its greatest concentration of diagnoses among those screened within the past six months, with progressive reductions across longer screening intervals.

**Figure 2:**
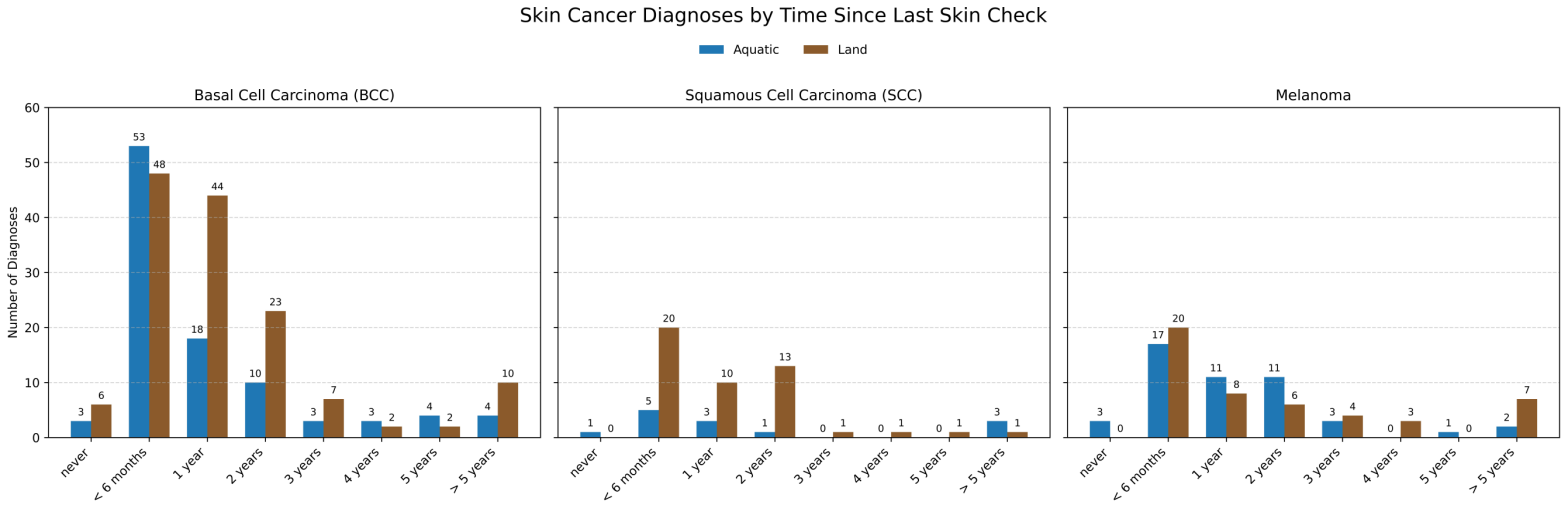
Temporal distribution of skin-cancer diagnoses relative to screening interval.

Melanoma (*n* = 96) and SCC (*n* = 48) exhibited markedly lower case counts overall, yet followed the same temporal trend in which the majority of confirmed diagnoses occurred among participants screened within the past two years. Although melanoma numbers were comparatively small, the highest frequency of cases was again identified in those reporting a recent (<six months, *n* = 37) or annual skin check (*n* = 19), with substantially fewer diagnoses emerging beyond the two-year interval. SCC showed the most concentrated clustering in the <6-month group (*n* = 25), with very few cases detected more than three years since last screening (*n* = 6) (Fig. 2).

### Multivariable logistic regression model

A multivariable logistic regression model was performed to identify factors associated with the likelihood of preferring a specialist clinician for skin checks. The full model contained nine predictors and showed no evidence of poor calibration (using the Hosmer–Lemeshow goodness of fit test (*χ*^2^ with eight degrees of freedom = 9.70, *p* = 0.29)). In the adjusted model, three variables were independently associated with increased odds of preferring a skin cancer doctor for skin checks. Participants with a prior skin cancer diagnosis had more than twice the odds of skin cancer doctor preference compared with those without a diagnosis (OR = 2.13, *p* < 0.001). Performing regular self or partner skin examinations was also associated with higher odds (OR = 1.61, *p* = 0.002), as was routine sunscreen use (OR = 1.49, *p* = 0.047). Age, gender, Fitzpatrick skin type, family history of skin cancer, total lifetime hours of UV-exposed activity type (aquatic *vs.* non-aquatic) were not statistically significant predictors (all *p* > 0.05).

## Discussion

### Overview of key findings

This study provides novel insights into skin-cancer screening practices, behavioral drivers, and clinician trust among a cohort of Australian outdoor enthusiasts exposed to high cumulative UV radiation (Harkensee & Hillebrandt, 2019). While many participants demonstrated positive engagement with both self-screening and clinical evaluation pathways, a significant minority, nearly 8%, had never received a professional skin check, despite elevated risk profiles due to prolonged UV exposure and a high prevalence of familial and personal skin cancer history. This behavioral gap highlights ongoing challenges in aligning public health recommendations with real-world practices in early skin cancer prevention (Janda et al., 2022). Importantly, the findings relate to patient perceptions of trust and preference rather than objective measures of clinical competence or diagnostic accuracy between provider groups.

Central to this cohort’s screening preferences was the role of clinician expertise. The overwhelming majority (84.2%) placed importance on who conducted their skin check, strongly suggesting that specialist trust is a key mediator of screening uptake. The high proportion of participants receiving their most recent skin check from a skin-cancer doctor likely reflects a combination of accessibility and perceived expertise in skin cancer assessment, aligning with our finding that trust in clinician capability is a dominant influence on screening choices. This pattern may reflect patient perceptions of expertise associated with different clinical settings, which have been discussed in prior literature comparing diagnostic practices across provider types. However, the present study did not evaluate diagnostic performance and therefore cannot determine whether differences in clinical outcomes exist between provider groups (Youl et al., 2007; Goldsbury et al., 2024). Notably, two-thirds of respondents had their most recent skin check performed by a skin cancer doctor, reinforcing the prominent role that this provider group plays in patient-reported screening pathways within Australian primary-care–based skin cancer services. (Margolis, 2019; Reyes-Marcelino et al., 2023).

### Trust, behavior, and the early detection pathway

Over half of participants in our cohort (54.9%) reported routinely conducting self- or partner-assisted skin examinations, a rate somewhat lower than national population benchmarks. The 2013–14 National Health Survey reported that two-thirds (66%) of Australian adults regularly self-checked for skin changes in moles or freckles (Australian Bureau of Statistics, 2015), and The Skin & Cancer Foundation’s 2017 survey similarly found that 70% of women and 64% of men performed routine self-examinations (Skin & Cancer Foundation Inc., 2017). These national estimates provide a useful comparator to our findings, suggesting that while self-screening was common among outdoor enthusiasts, its uptake was not markedly higher than the general population despite our participants having substantially elevated UV exposure. Importantly, 7.8% of participants had never undergone a professional skin check, indicating that a meaningful subgroup remains disengaged from formal screening pathways despite their high-risk profile.

Taken together, these patterns highlight a behavioral gap in which outdoor enthusiasts demonstrate moderate engagement with self-screening but inconsistent uptake of clinician-led checks, even though national recommendations emphasize both self-examination and regular professional surveillance for high-risk individuals (Cancer Council 2023a; Cancer Council 2023b; Royal Australian College of General Practitioners, 2025a; Royal Australian College of General Practitioners, 2025b). This gap is noteworthy given participants’ cumulative UV exposure, high prevalence of personal and family skin cancer history, and the recognized clinical importance of early detection. These findings collectively point to a need for enhanced patient education, risk communication, and system-level strategies to ensure that self-examination behaviors translate into appropriate, guideline-aligned clinical screening. If a high-risk subgroup is not meeting benchmarks for self-screening or skin checks, then one wonders how few lower-risk groups are performing these and raises a question regarding the current policy of self-examination and opportunistic screening.

Our findings suggest that trust in clinician expertise is not merely an abstract preference, but an actionable influence linked to pathway selection, particularly in high-risk, high-exposure populations. This aligns with contemporary patient-centred models of care, which emphasizes not only access to screening but also confidence in the clinician’s perceived expertise (Martin et al., 2024). In our sample, this translated to a predominant reliance on skin cancer doctors rather than GPs or dermatologists, potentially reflecting convenient access, lower cost, and perceived experience in lesion assessment and biopsy (Arasu, Meah & Sinclair, 2019; Coetzer-Botha et al., 2023).

The proportion of respondents using GPs for screening (20.9%) suggests opportunities for broadening skin-cancer-specific training and triage frameworks within primary care (Arasu, Meah & Sinclair, 2019; Reyes-Marcelino et al., 2023). Moreover, the proportionately lower referral to dermatologists may also reflect availability constraints in specialist services or adequate satisfaction with intermediate care providers (Goldsbury et al., 2024). These behavioral observations were subsequently tested in a multivariable logistic regression model to determine which variables independently influenced specialist screening preference.

The multivariable findings highlighted that personal disease experience was a far stronger determinant of screening preference than traditional risk indicators. Participants with a prior skin cancer diagnosis were more than twice as likely to favour skin cancer doctor-led screening. In contrast, family history of skin cancer, Fitzpatrick skin type, and cumulative UV exposure were not independently associated with the outcome after controlling for other factors. This pattern suggests that the lived experience of diagnosis exerts a stronger behavioral influence than perceived or inherited risk, reinforcing previous observations that risk awareness alone is often insufficient to modify screening behavior (Cust et al., 2011; Janda et al., 2014).

In addition, the results indicate a cluster of proactive health behaviors, where individuals who had already undertaken regular self or partner skin examinations and routinely used sunscreen were also more likely to prefer skin cancer doctor-conducted checks. This aligns with a broader prevention-oriented mindset rather than a single isolated behavior (Stapleton et al., 2016). Notably, activity context (aquatic *vs* non-aquatic) did not predict screening preference after adjustment, indicating that activity type, and by extension, differential UV exposure, is less influential than individual health history and behavior. Gender and age demonstrated near-significant trends, warranting further investigation in larger samples to clarify whether subgroup differences emerge with greater statistical power.

The clinic setting could suggest an inherent trust among attendees; however, meaningful variation in trust-based preferences remained evident. Multivariable analysis demonstrated that trust was not uniformly high but stratified by behavioral and experiential factors such as prior skin cancer diagnosis, routine self-examination, and sunscreen use. Because these patterns emerged statistically rather than descriptively, they indicate that trust in clinician expertise is not simply a by-product of attending a clinic but a measurable behavioral construct with real-world implications for provider selection and screening frequency.

### Implications for targeted screening among outdoor enthusiasts

Although aquatic participants demonstrated higher overall UV exposure, their screening behaviors and trust-based preferences did not differ significantly from those of non-aquatic peers, reinforcing the value of universal relevance of skin cancer doctor-led screening among outdoor participants, regardless of the specific outdoor activity context. However, the proportion of non-aquatic participants overdue for screening highlights an emerging subgroup that warrants further engagement and awareness strategies. Notably, the similar rates of self-examination between aquatic and non-aquatic participants suggest that a subset of outdoor enthusiasts may remain disengaged from formal screening pathways despite high cumulative UV exposure.

Given the alignment of our findings with national recommendations for risk-stratified, patient-led screening, future directions may include targeted clinician messaging, pop-up (Skin Check Champions, 2025) or mobile screening clinics at sporting events (Australian Skin Cancer Foundation, 2025), and norm-shifting communications that emphasize both the type of clinician involved and the frequency of checks aligned to UV exposure and personal risk history (Janda et al., 2022; Martin et al., 2024).

Recent national commentary has also recognized that Australia’s current approach to skin cancer screening may no longer be adequate for the country’s escalating burden of skin cancer disease (Cancer Council 2023a; Cancer Council 2023b). Several leading bodies, including the Melanoma Institute Australia (Melanoma Institute Australia, 2025), the Australasian College of Dermatologists (Australasian College of Dermatologists, 2024b), and the Australian Skin and Skin Cancer Research Centre (Australian Skin and Skin Cancer Research Centre, 2025), have called for a comprehensive review of national screening practices, citing inconsistencies in early detection pathways, variable diagnostic proficiency across clinician types, and the absence of a coordinated, risk-stratified framework. Contemporary analyses have similarly argued that opportunistic skin checks alone are insufficient and that system-level reforms, including clearer screening pathways and improved clinician training, may be required to ensure early and equitable detection of melanoma and keratinocyte cancers. These calls for review highlight a growing recognition that current models may not adequately meet the needs of high-risk groups, such as outdoor enthusiasts with substantial cumulative UV exposure.

### Diagnostic patterns and implications for screening frequency

Taken together, the observed temporal pattern, in which more diagnoses were identified among individuals reporting recent screening, is consistent with existing recommendations for regular surveillance. However, given the cross-sectional design, we cannot determine whether more frequent screening led to increased detection, or whether individuals at higher risk (*e.g.*, those with prior diagnoses, symptoms, or heightened concern) were more likely to undergo frequent screening.The markedly lower number of findings among individuals who had not been screened for several years may indicate either missed opportunities for earlier detection or lower engagement among those who perceive themselves to be at reduced risk. While our cross-sectional data cannot establish causality, the concentration of diagnoses within shorter screening intervals is consistent with existing recommendations for ongoing surveillance in high-risk groups and underscores the potential for diagnostic delay when follow-up intervals extend beyond recommended timeframes.

The temporal distribution of diagnoses observed in this cohort provides behavioral and clinical insight into patterns that are compatible with the potential value of regular screening. Across all three skin cancer types, the majority of confirmed cases were detected in participants who had undergone a skin check within the past 12 months, whereas markedly fewer diagnoses were recorded in those who had not been screened for several years. Taken together, this temporal pattern is consistent with established clinical understanding that lesions, particularly keratinocyte cancers, are more often identified when surveillance is recent, a pattern that may reflect the potential benefit of regular skin checks in high-exposure populations, although direct health benefit cannot be established in this study.

In both AK and BCC, the lowest number of diagnoses occurred in participants whose last skin check was at least ≥ five years ago, a pattern in which individuals reporting more frequent screening also had more lesions identified, although the directionality of this association cannot be determined. The notably low number of AK and BCC diagnoses among individuals whose last skin check was more than five years ago is consistent with the pattern that most lesions are identified in those undergoing more regular surveillance, although our data do not directly measure diagnostic yield or treatment uptake. This pattern is most pronounced for actinic keratoses and BCCs, which demonstrated steep declines in detection beyond the two-year interval, reflecting the typically high turnover and treatable nature of these lesions when surveillance is maintained.

The findings are particularly significant in relation to melanoma, where early detection is strongly associated with survival (Janda et al., 2022). Despite being the least frequent diagnosis in absolute terms, melanoma displayed the same detection clustering within the “recent screening” interval as keratinocyte cancers, with the highest number of cases identified in those screened in the previous 6–12 months. Very few melanomas were detected among participants who had gone more than two years without a skin check, which may indicate either a missed diagnostic window or the absence of clinically suspicious lesions being brought forward for evaluation. Given melanoma’s potential for rapid progression and metastasis, this temporal gradient is compatible with the clinical rationale for scheduled surveillance rather than symptom-triggered or opportunistic checks, although the present design does not permit inference of direct benefit.

Although our data do not directly measure diagnostic yield across different screening intervals, the wide variation in the time since participants’ most recent skin check highlights that many individuals may experience extended periods between examinations. In the context of established evidence showing that delayed melanoma diagnosis is associated with substantially higher morbidity and mortality compared with keratinocyte cancers, these prolonged intervals warrant careful clinical consideration. The results therefore support existing risk-stratified screening models that recommend at least annual professional examinations for individuals with high UV exposure, personal or family history of skin cancer, or phenotypic risk factors. More broadly, the alignment between recent screening and melanoma detection is consistent with existing screening recommendations; however, these findings should not be interpreted as demonstrating a direct causal benefit of increased screening frequency.

### Study strengths and limitations

This study’s strengths include its large and diverse sample of outdoor enthusiasts, which allows for meaningful comparisons between aquatic and non-aquatic groups. The survey captured both behavioral and clinical aspects of screening, including trust in clinician expertise, which adds depth to understanding drivers of screening behavior in high UV-exposed populations. Additionally, BCC, SCC and melanoma were confirmed *via* histopathology to confirm malignancy, considered the gold standard (Melanoma Research Alliance, 2024).

First, because all participants were recruited from a dedicated primary-care skin cancer clinic, a degree of selection bias is unavoidable. However, this setting does not invalidate behavioral insights: participants reported who performed their previous skin checks, and 36.4% had most recently been examined by a GP, dermatologist or plastic surgeon. These prior checks occurred across multiple settings, indicating that the distribution of past providers reflects genuine behavioral patterns rather than simply the effect of where participants were recruited. While attending a clinic implies a baseline level of trust in that environment, trust-related variation was still evident and statistically estimable across the cohort.

Recruitment through a clinical environment may overrepresent individuals who are more health-aware, more concerned about skin cancer, or already engaged in preventive care. Additionally, the absence of Fitzpatrick skin types I, V and VI likely reflects the demographic profile of the regional study population and clinic attendees rather than a true absence of these phenotypes within outdoor enthusiast populations more broadly. This distribution may limit generalizability to more ethnically diverse populations or geographic regions with different demographic compositions. Accordingly, these findings should not be extrapolated to all individuals who engage in outdoor activities without caution, particularly those who do not routinely seek clinical screening. Ideally, a broader sampling frame, including general practice and dermatology clinics, would have allowed a more comprehensive assessment of screening pathways; however, data collection is complete, and this approach was not feasible retrospectively.

Second, the terminology used to differentiate GPs, skin-cancer doctors, and dermatologists reflects patient self-report and perceptions rather than formal accreditation. Many clinicians working in skin cancer clinics also practise as GPs, and “skin-cancer doctor” is an unprotected title without standardized competency requirements. Accordingly, any preference expressed for this clinician type should be interpreted as reflecting perceived diagnostic expertise and the setting in which care is delivered, rather than as a verification of specialist status. Because participants elected to attend a dedicated skin cancer clinic, a baseline degree of trust in that setting can reasonably be presumed. Accordingly, preferences observed in this study reflect perceived trust and pathway choice rather than any verified differences in clinical competence or diagnostic performance between provider types.

Third, the cross-sectional design limits causal inference, and reliance on self-reported data introduces the potential for recall bias, particularly in relation to screening history and previous diagnoses. Prospective longitudinal research is needed to determine whether clinician trust, screening frequency, and provider choice lead to earlier-stage detection, reduced diagnostic delay, and improved clinical outcomes over time.

Despite these limitations, the study provides meaningful insight into behavioral drivers of screening in a high-UV-exposure population, including how trust, perceived expertise, and prior experiences shape real-world care-seeking decisions. These behavioral patterns remained robust in multivariable analyses, underscoring the relevance of these findings for understanding pathways to early detection.

### Conclusion

Outdoor enthusiasts, despite their elevated cumulative UV exposure, exhibited a mixed profile of proactive self-screening and lagging clinical engagement. Clinician trust emerged as a key determinant of where and how screening is conducted, with the role of specialized skin-cancer doctors central within this landscape. Future research should include longitudinal, prospective designs to determine whether screening frequency, clinician trust, and provider selection translate into earlier-stage diagnosis and improved clinical outcomes over time. Importantly, these findings reflect patient perceptions of trust and provider preference rather than objective differences in diagnostic capability. Such studies are necessary to clarify causal pathways and to evaluate whether the associations observed in the present cross-sectional cohort correspond to measurable health benefit.

## Supplemental Information

10.7717/peerj.21222/supp-1 Supplemental Information 1 STROBE checklist

10.7717/peerj.21222/supp-2 Supplemental Information 2 Raw data

## Additional Information and Declarations

### Data Availability

The following information was supplied regarding data availability: The raw data is available in the Supplemental File.
